# App-based learning for kindergarten children at home (Learning4Kids): study protocol for cohort 1 and the kindergarten assessments

**DOI:** 10.1186/s12887-020-02432-y

**Published:** 2020-12-08

**Authors:** Frank Niklas, Efsun Annac, Astrid Wirth

**Affiliations:** Department of Psychology, University of Munich (LMU), Leopoldstraße 13, 80802 Munich, Germany

**Keywords:** Home learning environment, Educational learning apps, Family intervention, Tablet-based learning, Kindergarten children, Home literacy environment, Home numeracy environment, Development of numeracy and literacy competencies, Digital learning, Mobile sensing

## Abstract

**Background:**

Children’s literacy and mathematical competencies are a critical platform for their successful functioning as individuals in society. However, many children, in particular those with low socio-economic status (SES) backgrounds who may not receive the home support needed to develop to their full potential, are at risk of not reaching sufficient competence levels. The overall aim of this project is to develop innovative computer tablet applications (‘apps’) and test whether the apps support parents in the provision of high-quality home learning environments (HLEs) and impact positively on the short- and long-term development of children’s competencies.

Altogether, “App-based learning for kindergarten children at home” (Learning4Kids) is a 5-year longitudinal study funded by the EU and designed to assess the potential impact of a tablet-based family intervention on children’s learning, development, social inclusion and well-being.

**Methods/design:**

This study uses a multi-method intervention approach and draws on expertise from psychology, education, informatics, and didactics to evaluate the effectiveness of learning apps and the intervention approach. It also exploits new technological possibilities afforded by tablet computers that are very common nowadays in families. Learning4Kids sets out to measure the quality of the HLE, children’s early mathematical, literacy, and cognitive competencies and their behaviour. Here, data will be gathered via standardized tests, observations, and parental and educator surveys and checklists. Data collection also includes the assessment of app usage times via mobile sensing. In cohort 1, 190 families are assigned to one of four groups. One business-as-usual group will only participate in the child assessments, whereas the three remaining groups are provided with tablets for about 10 months. Two intervention groups will receive mathematical or literacy learning apps as well as parental information about these topics and the tablet-control-group will receive similar apps and information that focus on general child development, but not on mathematics or literacy.

**Discussion:**

Whilst offering substantive advances for the scientific fields of psychology and education, the Learning4Kids study also has broad societal implications. Improving young children’s learning trajectories is both a social and economic imperative as it equips them to achieve greater individual success and to contribute to societal prosperity.

## Background

Children’s literacy and mathematical competencies are not only important prerequisites for academic achievement in school, but are also a critical platform for their successful functioning as individuals later in society. Formal teaching of reading, spelling, and mathematics begins when children enter primary school, however, children acquire abilities that are important for their later learning long before they enter school (cf. [1]). It is preferable to assess these skills early as these precursors are important predictors of academic performance in school (e.g., [2, 3]). For instance, early vocabulary predicts later literacy competencies and counting skills predict later mathematical competencies.

Given the importance of these precursors for children’s competencies development, child and family variables need to be identified that are associated with and influence these precursors and thus have an impact on later literacy and mathematical competencies. Research indicates that children’s individual characteristics such as intelligence, family background variables such as socioeconomic status (SES) and migration background, and interactions in the home learning environment (HLE) have an impact on children’s development of literacy and mathematical competencies (e.g., [1, 4, 5]).

Here, migration background and SES seem to influence students’ academic achievements indirectly rather than directly. According to Bradley and Corwyn [6], various explanatory factors such as differences in nutrition, living conditions, stress factors, parenting, parents’ educational aspirations or the HLE may play an important role. The influence of a child’s parents and family is not surprising, given that during the first few years of life, most children spend most of their time within the family context [7, 8]. In particular, the HLE seems to influence children’s early linguistic and numeracy competencies (e.g., [4, 5]) and child behaviour (e.g., [9, 10]).

### Children’s learning in a digital context

Given that nowadays, children in many countries worldwide grow up in media-rich homes and are in contact with a wide range of digital tools daily [11], these tools can be utilized to support children’s competencies development in many families, regardless of their backgrounds. This view also aligns with ecocultural theory as it acknowledges the important role of the immediate environment in young children’s learning (cf. [12]). Digital game-based learning uses the entertaining power of digital games to serve an educational purpose such as teaching math or language (cf. [13]). An explosion in available applications (‘apps’) has been noted over the last couple of years, especially for young children, and the majority of top-selling paid apps targeted young children [14].

However, many games and apps do not utilize developmentally-appropriate learning practices and/or have never been evaluated for their impact on child learning outcomes, and parents have difficulties to find and identify efficient apps among the numerous available apps in the app stores [14, 15]. Hirsh-Pasek and colleagues [15] described characteristics of efficient apps. In their view, educational apps for children need to be designed to promote

1. active,
2. engaged,
3. meaningful, and
4. socially interactive learning (four pillars).

Further, they conclude that children learn best when learning is also guided by a specific goal (i.e., the educational context of apps). Apps considering these aspects lead to deep learning and are thus highly likely to support children’s competencies development. Evidence that children learn from well-designed educational apps and e-books signals a new challenge to produce quality content and provide guidance for app developers [14].

### Rationale for the study

Whilst variables such as the family SES or migration background can hardly be changed, the HLE which is closely associated with these variables can readily be tackled. An improved quality in the HLE is very likely to impact positively on both, short-term and long-term child development and the HLE is thus the perfect target for interventions to improve child learning outcomes. Given the cost effectiveness of early intervention [16] and given that differences in the quality of the HLE and in early child competencies often persist from an early age [17–19], such interventions should be conducted well before children commence school.

Saracho [20] lists characteristics of successful interventions in the family context. Such interventions

1. use a multifaceted approach with families,
2. focus on directly stimulating and motivating the children,
3. educate the parents,
4. enrich and reinforce the quality of parent-child interactions and
5. enrich the caregiving environment (i.e., create a literacy/numeracy environment.

In fact, there are numerous examples of successful family interventions in the home environment (e.g., [21]). However, many studies have been conducted with medium to high SES families only and have not included many families with a migration background (e.g., [22, 23]), as for diverse reasons, low SES families seldom agree to participate in intervention studies [24]. However, children from low SES backgrounds and/or with a background of migration have a greater risk of academic failure (e.g., [25]) and the gap between children with low versus high SES widens over time [19]. Low SES families and families with a migration background should thus be prioritised for family interventions. One approach is to use the recent availability of digital tools and learning apps for children to support both, the quality of the HLE and children’s competencies development in all families.

### Study aims

Many children, in particular those with low SES backgrounds who may not receive the home support needed to develop to their full potential, are at risk of not reaching sufficient competence levels in literacy and mathematics [25]. Consequently, we need innovative, effective, easy-to-apply intervention approaches that focus on both, children’s early and later literacy and mathematical competencies and that also appeal to families who due to economic, educational, language or other constraints may shy away from extensive family interventions. Given that technical devices such as computer tablets are now available in most European households [11], apps offer a great opportunity to provide such interventions.

The overall aim of this project is to identify and develop innovative computer tablet applications (‘apps’) that not only provide important information about the HLE, effective math games and interesting children’s books, but also encourage participation in the intervention, remind families to actively improve the quality of the HLE and provide feedback. The apps will be tested to assess whether they support parents in the provision of high-quality HLEs and also impact positively on the short- and long-term development of children’s competencies. This randomized-control intervention project plans to analyse the development of children’s competencies in the years prior to and after school entry. Specific objectives of this project are to:

1. Identify, engage, and retain families with low SES and/or a background of migration in order that the intervention is completed.
2. Use non-intensive, easy-to-apply interventions to improve the quality of the HLE and consequently have a positive impact on children’s long-term learning trajectories
3. Use efficient tablet-based apps to (a) improve the quality of the HLE, (b) encourage parents and children to participate in the program and remind families to actively improve the quality of the HLE, (c) control the literacy and numeracy activities conducted with these apps, and (d) provide feedback on the usage times to parents.
4. Identify specific intervention approaches that focus on either the home literacy environment or the home numeracy environment and test whether these specific approaches influence the development of children’s literacy or mathematical competencies.

Today, we are living in the context of increasing heterogeneity in both family backgrounds and children’s early competencies and in a time in which new digital devices are readily available in households. Families differ greatly in regard to their available educational resources and the parental support they provide for children’s learning, and children of the same age will often have reached different levels of cognitive ability. We thus need family interventions that not only appeal to diverse families and children and encourage them to engage with this intervention, but that also improve the HLE and thus support children’s competencies development. This project will introduce such an intervention that follows the recommendations of Saracho [20]. Here, we will use apps with high pillar-scores according to the evaluation scheme of Hirsh-Pasek and colleagues [15] and evaluate the immediate and the long-term impact on both, families and children.

## Methods/design

**Fig. 1:**
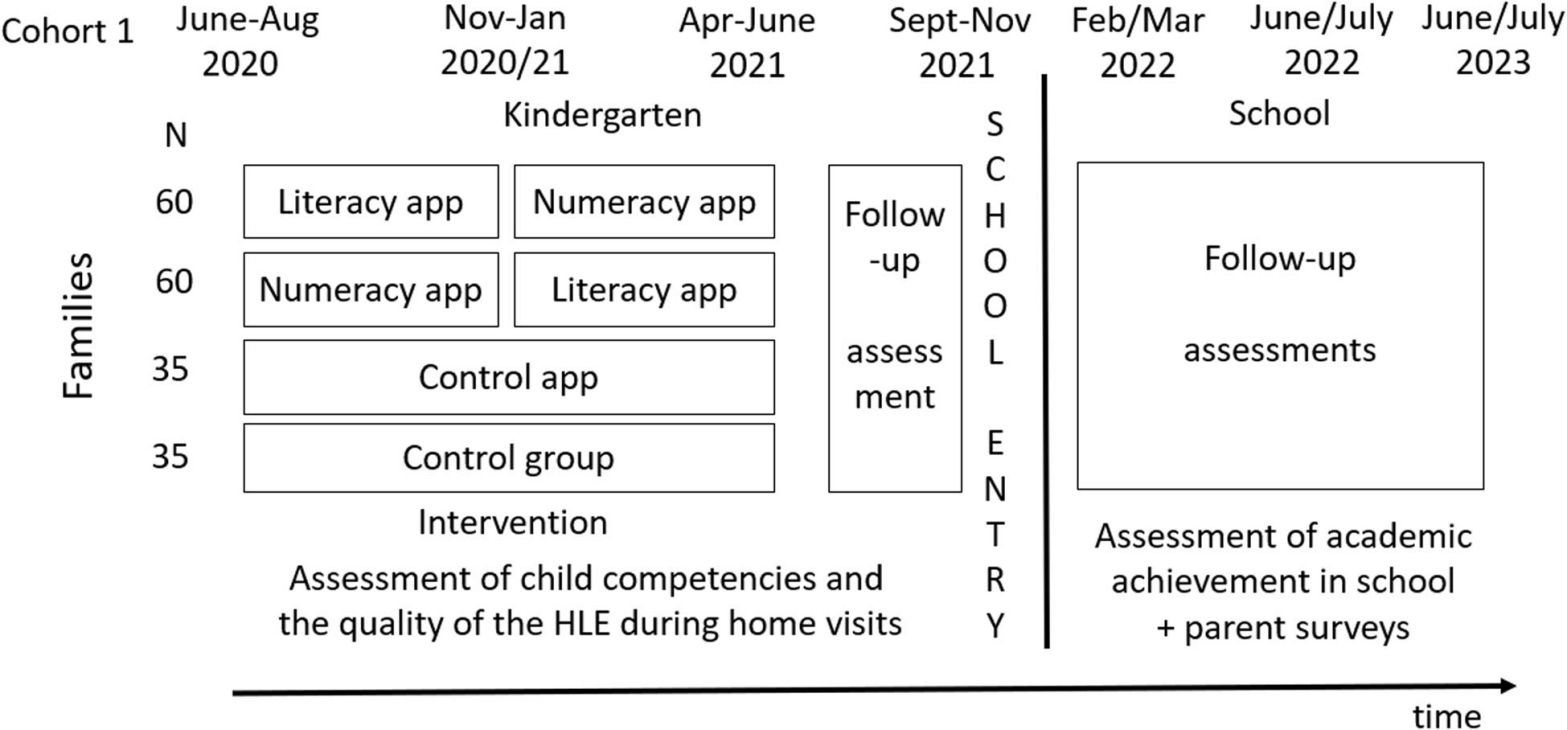
Schedule and timeline of the Learning4Kids project (first cohort)

The original plan in this study was to follow 500 families across four years from 1.5 years before school entry until the end of grade 2. However, the Covid-19 pandemic put an end to the recruitment in March 2020 and led to a later start with the assessments. Consequently, we decided to apply a two-cohort design instead, in which a second (younger) cohort will be recruited and will start the project at a later time. In the following, we introduce all information about the first cohort, the kindergarten assessments and the intervention approach that will also apply to the second cohort. The schedule for the assessments of cohort 1 is shown in Fig. 1.

### Ethics approval and consent to participate

For all research activities in this project, ethics is an integral part from beginning to end. In addition to the approval by the European Research Council Executive Agency, the thorough ethical evaluation also included ethical evaluation and approvals of the project by

1. The ethics committee of the Faculty of Psychology and Educational Sciences, University of Munich (LMU).
2. The department responsible for the implementation and management of research projects in public day care centres in Munich: The Department of Education and Sports, Munich.
3. The ministry responsible for the implementation and management of research projects in public schools in Bavaria: The Bavarian Ministry of Education.

Further, an external ethics advisor with the relevant independent expertise to monitor the ethical concerns in this project was appointed. She has provided, and continues to provide her advice on all relevant aspects of the study. All research actions in this project adhere to the ethical standards of data protection regulations according to Art. 13 EU-DSGVO. All participating families, childcare centre educators, directors of childcare centres, primary school teachers and primary school directors in this project were informed about these data regulations. Further, they were also informed that their participation is voluntary and that all data will be pseudonymised and published findings will be anonymised. All participants gave their written consent to these regulations of data handling and processing. Further, written and informed consent to participate in our study will be obtained from the parents of the participants in our study. Due to the COVID-19-pandemic, a research protocol was developed and applied for assessing children and their families with the best possible hygienic measure. The ethics advisor as well as the Department of Psychology at the University of Munich approved this document.

### Sample

The first cohort of our sample consists of *N* = 190 children with their families. Here, *N* = 60 families were randomly assigned to each of the experimental groups. In addition, *N* = 35 families were randomly assigned to each of the control groups with and without tablets. *N* = 114 participating families were recruited through the kindergartens attended by their child. In Germany, most children are enrolled in kindergartens from 2 to 3 years of age until the beginning of formal schooling at the age of 6 and almost all children attend kindergartens five days a week for several hours a day in the last two years before school entry [26].

We aimed to recruit a representative sample and thus lay a focus on families with a migration background and/or low SES. Here, statistical information from the ‘Indikatorenatlas’ for Munich was used to identify regions with high rates of unemployment and low income or in which a higher ratio of families with a migration background live (i.e., the child or at least one of the parents were born abroad). Kindergartens in these districts in the greater area of Munich were approached first, as most children attend kindergartens close to their homes [27] and thus many children in these kindergartens will often live in families with lower SES. The project was introduced and explained to kindergarten directors and educators via telephone calls. In participating kindergartens, plain language statements about the project and consent forms were provided to parents in the most commonly used foreign languages of the families (e.g., Turkish, Polish, Russian, Albanian, Romanian, Arabic, Persian (farsi), Spanish, Italian, Vietnamese, and English). In a second step, families from the greater Munich area were contacted via a social media campaign on facebook. Here, potential participants were approached via telephone calls by a market research institute, introducing and explaining the project to interested families. After families signed the consent form, the kindergartens attended by their participating child were contacted and introduced to the project, too. With this approach, *N* = 71 additional families were recruited. Five additional families were recruited by word of mouth and leaflets distributed in the city. Altogether, the participating children attended *N* = 111 different kindergartens. In each participating kindergarten, between *N* = 1 and *N* = 11 children (and their parents) participated in our study. Descriptive statistics for all study groups are presented in Table 1.

**Table 1:** Descriptive statistics for the four groups of participating families

	Tablet group 1 (Literacy)	Tablet group 2 (Numeracy)	Control group 1 (Control tablet)	Control group 2 (No tablet)	Total sample
Number of participants, *N*	60	60	35	35	190
Children‘s age at t1 in months, *M (SD)*, range	63.4 (4.0) 55–73	62.9 (4.3) 54–75	63.7 (5.0) 51–73	65.0 (4.4) 53–73	63.6 (4.4) 51–75
Children’s sex: male / female	28 / 32	31 / 29	17 / 18	16 / 19	92 / 98
Families with migration background^a^, *N* (%)	26 (43.3)	23 (38.3)	17 (48.6)	14 (40.0)	80 (42.3)
SES^b^	98.2 (34.8)	91.2 (37.3)	90.0 (40.6)	89.4 (32.1)	92.8 (36.1)

Notes: ^a^migration background: At least one parent or the child being born outside of Germany;

^b^SES, highest family occupational prestige [28], however preliminary prestige score only (*N* = 168) due to missing data and unspecific answers

No significant differences between groups were found for the descriptive statistics. The ratio of families with a migration background was higher than in the general population of this age group [29], indicating that the recruitment plan had worked. On the other hand, the average highest prestige score in a household was similar to other studies with medium to high SES samples (e.g., [22]). However, this might change once the missing data has been assessed at t2.

### Measures

Assessments in Learning4Kids included educator and parental surveys and checklists, observations and interviews in the family, and child test assessments (for an overview, see Table 2).

**Table 2:** Overview and summary of variables assessed in Learning4Kids

Level	Domain	Name (short form)	Method	Cronbach’s α	Variable details / Subtests
Educator	Socioemotional development	EBD	Survey	.82^a^ /.84^a^	Milestones in children’s social and emotional development
	Problematic behaviour	SDQ	Survey	.66 ^a^ /.70^a^ / .86^a^ /.64^a^ /.76^a^	Subscales: Emotional symptoms, conduct problems, hyperactivity/inattention, peer relationship problems; prosocial behaviour
	Attention deficit hyperactivity disorder	Conners’ scale	Survey	.85^a^	ADHD Index; short form
	General child competencies	-	Survey	-	Concentration, language skills, vocabulary, grammar, mathematics skills, motor skills, social and emotional competencies, logical thinking
	General child development	-	Survey	-	Age-appropriateness of child’s development, school readiness, special needs
	Media usage	-	Survey	-	Number and usage of different media in the kindergarten (e.g. books, games, tablets)
	Social popularity	-	Survey	.74^a^	Acceptance, rejection and disregard by other children in the kindergarten
	Kindergarten attendance	-	Survey	-	Start of kindergarten attendance, daily attendance in hours
Family	Home Literacy Environment	HLE	Survey	.79-.89	Shared reading, linguistic activities, literacy exposure, literacy instructions, formal HLE
	Home Numeracy Environment	HNE	Survey	.80-.83	Mathematical activities, number / mathematics exposure and instructions, formal HNE
	Book title recognition	TRT	Checklist	.87	Checklist on selected children’s book titles, mixed with fake distractor titles
	Math game title recognition	MTRT	Checklist	.69	Checklist on selected children’s mathematical game titles, mixed with fake distractor titles
	Educational aspiration	-	Survey	.64	Parental educational aspiration for child
	Media usage	-	Survey	-	Number of different kinds of media at home, children’s and parental media usage
	Movie watching	-	Survey	.74	Parental and child video and movie watching during the week and on weekends
	Attitudes towards digital media	-	Survey	.75	Parental attitudes towards media
	Media literacy	-	Survey	.81/.86	Children’s and parental media literacy
	Family background	-	Survey	-	Migration background of parents and child^1^, spoken languages at home, educational background of parents, occupation of parents^2^, monthly income, number and age of siblings, family living conditions
	Learning disabilities	-	Survey	-	Family history of dyslexia, dyscalculia, AHDH
	Shared reading	FES adapted^3^	Observation of shared reading	.84	Verbal dissociation, nonverbal behaviour, use of questions, explanations of letters/words, phonological explanations, free language level, balance of conversation (child / parent)
	Dice game play	FES adapted^3^	Observation of game play	.87	Counting, nonverbal behaviour, mentioning of numbers, free language level, explanations of mathematical concepts, comparing / ordering, references to everyday life
	Parents’ behaviour and living conditions	HOME adapted^4^	Observation in the family	.64 / .46 / .69	Parents’ behaviour towards the child, parents’ attitudes towards the child (affection and warmth), families’ physical environment and living conditions
	Miscellaneous parent-child interactions	HOME adapted^4^	Interview	-	Number of children’s games, frequency of museums visits, teaching of colours and scales, communicativeness of the child, physical contact with the child, TV usage
Child	Mathematical competencies	MARKO-S	Test	.82	Numbers, ordinal number bars, cardinality, number division, inclusion and relations.
		WVT/ number sequence forwards	Test	.79	Number sequences forwards
		WVT/ number sequence backwards	Test	.70	Number sequences backwards
		WVT/ predecessors and successors	Test	.81	Number predecessors and successors
		WVT/ Numerical knowledge	Test	.87	Number symbol knowledge, knowledge of numeric representations
		MBK-0; Calculating task	Test	.78	Mathematical multiplication and subtraction
	Linguistic competencies	AWST-R (verbs and nouns)	Test	.65	Active / expressive vocabulary
		PPTV	Test	.95	Passive / receptive vocabulary
		WVT; rhyming task	Test	.79	Rhyming words, phonological awareness
		WVT; letter sound identification	Test	.88	Identification of first letter sound, phonological awareness
		WVT; letter knowledge	Test	.88	Productive letter knowledge
		WVT; letter knowledge	Test	.79	Receptive letter knowledge
		SETK 3-5; plural forms	Test	.82	Plural forms of (non-)words, grammar
		EuLe 4-5; early literacy	Test	.79	Early literacy knowledge, concept of reading and writing
	Intelligence	CMMS	Test	.92-.96^b^	Short nonverbal intelligence test, general reasoning ability, abstract thinking
	Concentration	KKA	Test	.88-.98^c^	Short-term selective attention and concentration
	Working memory	WVT; working memory	Test	.80^d^	Digit span working memory task -forward
	Rapid naming	-	Test	-	Rapid automatized naming of objects/pictures
	Self-concept	BSLS-M	Test	.72	Mathematical self-concept
		BSLS-S	Test	.75	Literacy self-concept
		BSLS-pro	Test	.69	Prosocial self-concept
		BSLS-anti	Test	.82	Antisocial self-concept
	Prosocial behaviour	-	Test	.78	Prosocial behaviour, sharing

Notes: ^1^Migration background: At least one parent or the child being born outside of Germany

^2^SES: highest family occupational prestige [28]

^3^FES: adapted version of the “Family rating scale” ([30], cf. [31])

^4^HOME: selected items from the “Home Observation for Measurement of the Environment” (cf. [31, 32])

^a^Preliminary reliability index (*N* = 119) due to on-going assessment

^b^CMMS split-half reliability according to Esser [33]

^c^KKA reliability according to Krampen [34]

^d^WVT; working memory reliability according to Endlich et al. [35]

#### Educator survey

Kindergarten educators were asked to fill in a survey on children’s characteristics. The educators rated the media activities in kindergarten such as which digital tools are available in the kindergarten and how often they are used. Further, the educators were asked to provide a general evaluation about children’s concentration, linguistic, mathematical and socio-emotional competencies. Here, a focus lay on children’s emotional and social abilities (assessed with the EBD [36]), on children’s behavioural strengths and difficulties (Strengths and Difficulties Questionnaire, SDQ [37], and symptoms of attention-deficit-hyperactivity-disorder (short form of the Conners’ rating scale [38, 39] (see Table 2).

#### Parental survey

As with the Plain Language Statements, parental surveys were provided in several languages addressing families with various migration and language backgrounds (see above). Parents were asked questions on the family background, children’s characteristics and the HLE [4, 32, 40]. The focus lay on various aspects of the HLE and the first part assessed the home literacy environment with items on linguistic activities and literacy exposure of the study child at home (example items: “how many books do you have at home”, “how often is your child read to”). In the next part of the survey, parents were asked questions about the home numeracy environment (example items: “How often do you play games with your child that require him or her to count?”, “How often does your child participate in counting or measuring ingredients when cooking?”). In addition, two checklists were used to get further insight into the home literacy and numeracy environment. Here, a German book title recognition test for children’s books (TRT [41]) and a German mathematical game titles recognition test (MTRT [42]) were applied. Selected titles of children’s books and math games were mixed with fake book and game titles to objectively assess parents’ knowledge on these books and games.

Further, parents’ expectations for their child’s future school education (example item: “The education of my child in school is important to me”), children’s and parents’ media usage (example item: “Do you have learning apps/games for children on your tablet/smartphone? How often are these used?”), attitudes towards digital media (example item: “Digital media for preschool children are important”), and children’s and parental media literacy (example item: “How would you rate your ability to find appropriate media content for children?”) were assessed. Finally, parents were asked about the family background. Here, items on migration background, SES including the educational and occupational background of both parents and the family income, and on learning disabilities in the family had to be answered (see Table 2).

#### Assessment of the HLE

Different studies have used different operationalizations to assess the HLE. Sometimes the HLE was measured directly in the family by surveillance of the learning environment a family provides (e.g., [43]), or questionnaires were used in which the parents were asked to indicate their knowledge about children’s books (cf. [41]). Most studies, however, and in particular large-scale studies, used surveys and asked the parents, for example, about the number of books and picture books in the household, how often they read to their children, or how often they played numeracy-based games with their children (cf. [32, 40]). In Learning4Kids, a combination of all these measures (survey and interview items, checklists, and observations) is applied (see Table 2).

#### Test instruments

Several test instruments are used to assess various child competencies (for an overview including Cronbach’s alphas, see Table 2). During the project period, these assessments will be conducted at four different time points in the kindergarten and early school phase. The first assessments (t1) in summer 2020 included the test instruments below:

1. Mathematical competencies.

Several mathematical and arithmetic (sub)tests for kindergarten children were used to assess children’s mathematical competencies. For instance, these tests addressed children’s counting, calculating, comparing of numbers and amounts, and number knowledge. Here, the screening “Mathematik- und Rechenkonzepte im Vorschulalter-Screening” (MARKO-S; [44]), different subtests from the “Würzburger Vorschultest” (WVT; [35]) and an adapted version of the calculation subtest of the “Test mathematischer Basiskompetenzen im Kindergartenalter” (MBK-0; [45]) were applied (see Table 2).

2 Linguistic and literacy competencies.

Children’s linguistic and literacy competencies were also assessed with various (sub)tests. We assessed both, children’s active vocabulary with 15 items from the “Aktiver Wortschatztest für 3- bis 5-jährige Kinder – Revision” (AWST-R [46]) and children’s passive vocabulary with nine sets from the German version of the “Peabody Picture Vocabulary Test” (PPVT [47]). Further, subtests from the WVT [35] assessed children’s phonological awareness with a rhyming task and an initial sound analysis task as well as the active and passive letter knowledge. In addition, in a grammar test from the “Sprachentwicklungstest für drei- bis fünfjährige Kinder“ (SETK 3–5 [48]), children’s ability to build plural forms of words and non-words was tested. Finally, the subtest “Schriftwissen” from the test “Erzähl- und Lesekompetenzen erfassen bei 4- bis 5-jährigen Kindern“ (EuLe 4–5 [49]) assessed children’s early literacy knowledge and knowledge about reading.

3 General cognitive abilities.

In addition to the numeracy and literacy assessments, children’s general cognitive abilities were tested with several test instruments. The Columbia Mental Maturity Scale (CMMS [50]) was used to assess children’s nonverbal intelligence. Further, children’s ability to concentration was assessed with the “Kaseler-Konzentrations-Aufgabe für 3- bis 8-Jährige” (KKA [34]). Children’s working memory capacity was measured with a forward digit span task (a subscale of the WVT [35]). Finally, a rapid picture naming task (cf. [51]) was used to test children’s fast access to long-term memory.

4 Self-concept.

Children’s academic, prosocial, and antisocial self-concept were assessed with an adapted German version of the “Pictorial Scale of Perceived Competence and Acceptance for Young Children” (PSCA, in German BSLS; [52, 53]). Here, children self-rated their early mathematical and literacy abilities. In addition, they were asked to rate their potentially prosocial and antisocial behaviour in different situations that were described to them and shown on pictures.

5 Prosocial and punishing behaviour.

Finally, children were told a fictitious situation, in which they had the possibility to share small gifts such as rubbers, stars or stickers with another child who was shown to them on a picture and who in the story did not get such items yet. In addition, they were also asked, whether their own sharing behaviour was good and “why” or “why not” it was good, and whether another fictitious child who did not share should be punished (cf. [54–56]).

### Intervention approach

The intervention in Learning4Kids uses a tablet-based approach. For this intervention, already existing learning apps were identified, and new ones were designed and developed that are suited to promote efficient learning of kindergarten children at home. Here, the recommendations of Hirsch-Pasek and colleagues [15] and the suggestions of Saracho [20] were considered during the whole process (see below).

In our randomized-control-group design, children and their families were assigned to either one of two intervention groups or one of two control groups (one group with a tablet and the other one without tablet as a ‘business-as-usual’ group). The two intervention groups received tablets with literacy and numeracy apps in counterbalanced order (i.e. one group started with the literacy apps while the other group started with the numeracy apps, and after the first intervention phase of about five months the apps will be swapped). The tablet-control group received tablets with control apps that do not focus on literacy and numeracy (see Table 4).

In addition to the learning apps, children also received e-books, audio books, and children’s music. Whereas the numeracy intervention group received five e-books and one audio book that focused on numbers, the control group received two e-books for children on emotions and social interaction, one audio book on concentration, and three music CDs for children and in the second intervention phase, two e-books, three audio books and one music CD. In comparison, the literacy group received altogether 23 e-books, 32 audio books, and 7 music CDs and videos for children as shared reading and listening to audio books is a specific and key part of family literacy interventions. To balance the input between the three tablet groups, the literacy group received only 12 instead of 18 learning apps during the intervention.

The third part of the intervention focused on parents and will be described in the following.

#### Parental information and tips

Parental information and tips were provided for all tablet groups. Here, various topics on child development and recommendations for the interaction with children at home were addressed (see Table 3). The parental information for the literacy group focussed on five early literacy topics, whereas the parental information for the numeracy group focussed on five early numeracy topics. The control group received information about ten topics on general child development. Every month, a new parental information (about 1600 words) was provided and within each of the parental information documents, also three different scientific studies or theories were described in an easy-to-understand language.

**Table 3:** Overview of the parental information and the tips provided to families

		Parental information (Literacy / Numeracy / Control1 / Control2)	Tips
Month 1	Week 1	Verbal interactions & modeling /	Lit/Num/Con1
	Week 2	Mathematics in everyday life /	Lit/Num/Con2
	Week 3	Motoric development /	Lit/Num/Con3
	Week 4	Development of musical skills	Lit/Num/Con4
Month 2	Week 5	Shared reading /	Lit/Num/Con5 +1
	Week 6	Numbers and parents as role models /	Lit/Num/Con6 +2
	Week 7	Emotional development /	Lit/Num/Con7 +3
	Week 8	Prosocial & moral development	Lit/Num/Con8 +4
Month 3	Week 9	Rhyming and word games /	Lit/Num/Con9 +5
	Week 10	Counting and comparing /	Lit/Num/Con10 +6
	Week 11	Social development /	Lit/Num/Con11 +7
	Week 12	Memory and logical thinking	Lit/Num/Con12 +8
Month 4	Week 13	Library use & children books /	Lit/Num/Con13 +9
	Week 14	Mathematical games /	Lit/Num/Con14 +10
	Week 15	Concentration and attention /	Lit/Num/Con15 +11
	Week 16	Self-confidence & self-esteem	Lit/Num/Con16 +12
	Week 17		Lit/Num/Con17 +13
Month 5	Week 18	Learning to read and write /	Lit/Num/Con18 & 21 +14
	Week 19	Measurement, forms, and teaching by the parents /	Lit/Num/Con19 & 20 +15
	Week 20	Personality and temperament /	Lit/Num/Con16, 18 & 20
	Week 21	Creativity development	Lit/Num/Con17, 19 & 21

Note: *Lit* Literacy, *Num* Numeracy, *Con* Control group tips number 1 to number 21

Further, every 8 days (in Learning4Kids a “study week” lasted 8 days, so that each intervention phase lasted at least 5 months for each family), parents received easy-to-apply tips according to the topic of the parental information they received for their everyday interaction with their child (examples for the literacy, numeracy, and control group: “Is your child bored because you have to wait? Let your child name all the objects he or she can see at that moment! (e.g. a chair, a plant, a dog ...)”; “Use mealtimes for “mathematical interactions”: Ask your child to set the table, and count cutlery and dishes. Compare food on the plates: which food is “more” or “less"”; “Take a photo of physical activities and create a photo book for every month or every year. Talk to your child about the activities your child enjoys the most and make plans together, so that your child can do these activities regularly”). Each of the 21 tips for each group was provided to the parents during two different study weeks (e.g., tip 1 was provided in week 1 and then again in week 5 together with tip 5; see Table 3). The tips for parents included suggestions for family activities at home and in everyday situations to improve the quality of the HLE and support children’s literacy, mathematics or general development independent of the tablet usage (see Table 3).

#### Learning-apps for children

One aim of our tablet intervention was to develop educational and engaging apps to promote efficient learning and support children’s academic competency development. For the intervention group, we designed educational and interactive apps that target early mathematical and early literacy skills and for the control group, we designed apps targeting general cognitive functions with no literacy and mathematical content. If possible, apps were developed as similar as possible, that is, games were planned for all three groups but different stimuli were used depending on the group. For instance, a memory game app was provided to all groups. In the literacy group, memory cards were presented with letters and in combination with animals whose names start with a matching letter; in the numeracy group, numerical values in different forms such as dots on dices, fingers of a hands or Arabic numbers were used, and the control group received a colours memory game in the first intervention phase and an animal memory game in the second intervention phase. With this approach, we tried to keep the input as similar as possible for all groups.

However, this was not possible for all apps, as some apps focused on very specific content such as “Find the vowels”, “Sentence understanding”, “Measurement”, and “Learn the clock”. As with the e-books, audio books and parental information, each month new apps were added to keep the children and their families interested.

Literacy apps focused on linguistic development with activities such as letter learning, letter drawing and sorting, rhyming, phonological awareness, and word and sentence understanding. Mathematical apps included numerical activities such as number learning, number drawing and sorting, counting, measurement and learning the clock. Control apps included games based on sorting, drawing, and learning shapes and colours, puzzles, and activity games (see Table 4).

**Table 4:** Overview of the learning apps used in Learning4Kids intervention

		Literacy intervention	Numeracy intervention	Tablet-control group
	App 1	Memory (Letters)	Memory (Numbers)	Memory (Colours)
	App 2	Letter drawing	Number drawing	Line drawing (Labyrinth)
Month 1	App 3	Painting with letters	Painting with numbers	Painting with colours
	App 4	Letter sorting	Number sorting	Animal puzzle
	App 5		Build a number rocket	Build a colour rocket
	App 6	Initial letter sounds	Collecting nuts (numbers)	Collecting nuts (Colours)
	App 7	Snakes & Ladders (Letters)	Snakes & Ladders (Numbers)	Snakes & Ladders (Colours)
Month 2	App 8		Tap it! Numbers	Tap it! Animals
	App 9		Mathemarmite	Sagomini Forest Flyer
	App 10	Find the vowels	Measurement app	Sagomini Friends
Month 3	App 11	Finding pairs (rhymes)	Finding pairs (numbers)	Finding pairs (colours)
	App 12		Count and compare	Bird Tower
	App 13	Sentence understanding	Counting the ballons	Fish-Maze
Month 4	App 14	Letter-Domino	Number-Domino	Colour-Domino
	App 15		Connect the number dots	Connect the colour dots
	App 16	Word-Puzzle	Learn the clock	Animal Maze
Month 5	App 17	Magic potion (sounds)	Count and sort objects	Piano app
	App 18		Categorize numbers	Categorize colours

#### Intervention fidelity

Intervention fidelity refers to the extent to which core components of interventions are delivered as intended by the protocols [57]. However, fidelity of implementation is rarely reported in large-scale education studies that examine the effectiveness of kindergarten or school core curriculum interventions, especially with regard to how fidelity enhances or constrains the effects of the intervention on outcomes [58].

As recommended by Gearing and colleagues [57], we prepared elaborate, detailed intervention protocols, provided a comprehensive assessment training for all team members and guidelines for the parents how to use the tablets, and monitored the intervention delivery via a checklist and intervention receipt and fidelity via an app on the tablets (“Phone study app”, PSA). In addition, feedback can be used to strengthen intervention fidelity [57] and therefore, families also receive a weekly feedback on their usage time as measured by the pre-installed PSA on their tablets. Here, the PSA uses “mobile sensing” (cf. [59]) to assess app usage times of children and their parents. Further, the PSA is linked to a reward system for children that is based on their actual app usage times (e.g., for every 30 minutes of total app usage a new reward is provided to children).

Our general intervention approach will thus support intervention fidelity and parental commitment to the project for its duration (cf. [57]). Further, our intervention approach also includes home visits and thus overcomes typically occurring barriers in family intervention research such as low attendance of family intervention sessions, low parent engagement during the entire session, and low return rate of surveys and assessments (e.g. [60]).

### Analytic strategy

The analytical plan will include both, intention-to-treat analyses (i.e., comparison of the treatment and control groups that includes all children and families as originally allocated after randomization) and per-protocol analyses which in the case of this study will take into account how often specific apps had actually been used in the intervention groups. Further, analyses will cover potential immediate post-intervention effects as well as potential long-term effects on school achievement.

## Discussion

One important challenge for policy and research is to find ways to support the development of children who start life disadvantaged due to their family circumstances and the lower quality home learning environment they experience. Family background variables such as SES or a migration background can hardly be changed, but the HLE, which is closely associated with these variables, can readily be tackled.

This project exploits new technological possibilities afforded by smart phones and tablet computers that are very common nowadays in families and that will appeal to many families, regardless of their backgrounds. Whilst countless so-called ‘learning games’ and ‘learning apps’ for children are available online, few have been evaluated. The planned intervention will lead to empirically tested, inexpensive software for interested families that will be accessible to the general public due to low download fees as well as scope to translate the apps into other languages.

In addition to the learning apps, our intervention also includes parental information and tips for everyday interaction with their children independent of any technical devices. Consequently, we follow a multifaceted approach with families and focus on directly stimulating and motivating the children as well as educate the parents. Altogether, we believe that our intervention approach will enrich and reinforce the quality of parent-child interactions and enrich the caregiving environment [20].

### Conclusions and potential impact

With this intervention, large numbers of families and children could be accessed and it might impact positively on several important areas:

1. the education of all children whose learning may be supported by meaningful and well-developed learning apps,
2. the integration of children who start life disadvantaged by their family circumstances, which influences later individual success and the opportunities to fully develop their potential and contribute to societal prosperity,
3. the support of parents who yet have to fully embrace the possibilities of smartphones and tablet computers in the context of education as they might shy away from learning apps that have not been positively evaluated,
4. substantive advances for the scientific fields of psychology and education.

Therefore, we believe that this project has the potential to not only provide us with important scientific insight, but also to have a positive impact on child development, families, and the broader society.

## Data Availability

The raw data supporting the conclusions of this manuscript will be made available by the authors, without undue reservation, to any qualified researcher on reasonable request.

## Abbreviations

Apps: Computer tablet applications
AWST-R: Active vocabulary test for 3- to 5-year-old children
CMMS: Columbia Mental Maturity Scale
EBD: Observation and documentation of children’s development 48–72 months
ERC: European Research Council
EuLe 4–5: Assessment of narrative and reading competencies of 4- to 5-year-old children
HLE: Home learning environment
KKA: Kaseler-Concentration-Task for 3- to 8-year-olds
Learning4Kids: App-based learning for kindergarten children at home
MARKO-S: MARKO-Screening – mathematics and concepts of calculation before school entry
MBK-0: Assessment of basic mathematical competencies in kindergarten age
MTRT: Mathematical game titles recognition test
PPVT: Peabody Picture Vocabulary Test
PSA: Phone study app
PSCA: Pictorial Scale of Perceived Competence and Acceptance for Young Children (BSLS)
SDQ: Strengths and Difficulties Questionnaire
SES: Socio-economic status
SETK 3–5: Linguistic development test for 3- to 5-year-old children
TRT: German book title recognition test for children’s books
WVT: Würzburg preschool test:Assessment of literacy and mathematical (precursor) abilities and linguistic competencies in the last year of kindergarten
